# Risk estimates of recurrent congenital anomalies in the UK: a population-based register study

**DOI:** 10.1186/s12916-017-0789-5

**Published:** 2017-01-31

**Authors:** Svetlana V. Glinianaia, Peter W. G. Tennant, Judith Rankin

**Affiliations:** 1 Institute of Health & Society, Newcastle University, Baddiley-Clark Building, Richardson Road, Newcastle upon Tyne, NE2 4AX UK; 2 School of Healthcare, University of Leeds, Baines Wing (Room 1.11), Leeds, LS2 9JT UK

**Keywords:** Congenital anomalies, Northern Congenital Abnormality Survey (NorCAS), Recurrence, Prenatal counselling, Siblings

## Abstract

**Background:**

Recurrence risks for familial congenital anomalies in successive pregnancies are known, but this information for major structural anomalies is lacking. We estimated the absolute and relative risks of recurrent congenital anomaly in the second pregnancy for women with a history of a congenital anomaly in the first pregnancy, for all major anomaly groups and subtypes.

**Methods:**

Population-based register data on 18,605 singleton pregnancies affected by major congenital anomaly occurring in 872,493 singleton stillbirths, live births and terminations of pregnancy for fetal anomaly were obtained from the Northern Congenital Abnormality Survey, North of England, UK, for 1985–2010. Absolute risks (ARs) and relative risks (RRs) for recurrent congenital anomaly (overall, from a similar group, from a dissimilar group) in the second pregnancy were estimated by history of congenital anomaly (overall, by group, by subtype) in the first pregnancy.

**Results:**

The estimated prevalences of congenital anomaly in first and second pregnancies were 275 (95% CI 270–281) and 163 (95% CI 159–168) per 10,000 respectively. For women whose first pregnancy was affected by congenital anomaly, the AR of recurrent congenital anomaly in the second pregnancy was 408 (95% CI 365–456) per 10,000, 2.5 (95% CI 2.3–2.8, *P* < 0.0001) times higher than for those with unaffected first pregnancies. For similar anomalies, the recurrence risk was considerably elevated (RR = 23.8, 95% CI 19.6–27.9, *P* < 0.0001), while for dissimilar anomalies the increase was more modest (RR = 1.4, 95% CI 1.2–1.6, *P* = 0.001), although the ARs for both were 2%.

**Conclusions:**

Absolute recurrence risks varied between 1 in 20 and 1 in 30 for most major anomaly groups. At pre-conception and antenatal counselling, women whose first pregnancy was affected by a congenital anomaly and who are planning a further pregnancy may find it reassuring that, despite high relative risks, the absolute recurrence risk is relatively low.

## Background

Congenital anomalies are a leading cause of morbidity and mortality in early life. Affecting 1–6% of viable pregnancies worldwide [1–4], they cause around 3.3 million annual deaths in children aged under 5 years [3], including a quarter of all infant deaths in high income countries [5–7]. For long-term survivors, the prognosis varies greatly between conditions and settings, but many experience significant physical and/or psychological impairments, resulting in sustained health and social care needs at considerable cost [8].

Although the number of children born with the most severe congenital anomalies has reduced with improvements in the availability and sensitivity of prenatal screening [1, 9], a congenital anomaly diagnosis, and the subsequent discussion around termination of pregnancy, is associated with significant emotional distress [10]. Families with a familial condition or who have previously lost a child or pregnancy to a congenital anomaly are hence often particularly concerned about the risk of recurrence in future pregnancies [11].

Genetic counselling provides guidance and support for those families who are affected by conditions with known inheritance patterns [12, 13], but since the aetiology of most congenital anomalies is multifactorial or unknown [14], there is a lack of information concerning the recurrence risks for most anomaly groups and subtypes. The best available data come from three population-based studies published in the 1990s, all of which found that previous congenital anomaly was associated with around twice the risk in a subsequent pregnancy, including five to twelve times the risk for similar anomalies [15–17]. Unfortunately, modest sample sizes, the use of outdated and unclear classification schemes (e.g. including a high proportion of minor anomalies) and a lack of detail for specific congenital anomaly subtypes limit their value for current pre-conception and prenatal counselling.

This study used data from the UK’s longest-running population-based register of congenital anomalies to estimate the absolute and relative risks of recurrent congenital anomaly in the second pregnancies of mothers with a history of congenital anomaly in their first pregnancy.

## Methods

### Study population

The Northern Congenital Abnormality Survey (NorCAS) records details of all cases of congenital anomaly whether arising in late miscarriage (20–23 weeks’ gestation), termination of pregnancy for fetal anomaly (TOPFA) following prenatal diagnosis (any gestation), stillbirth (≥24 weeks’ gestation) or live birth to mothers resident in the North of England (population: ≈3 million; births: ≈32,000 per year). The North of England is characterised by a relatively stable population with low levels of both inward and outward migration, and a relatively low percentage (about 5%) of the population is from minority ethnic groups [18]. Data on all major congenital anomalies occurring in singleton pregnancies to women resident in the region from 1 January 1985 to 31 December 2010, regardless of place of delivery, were obtained from the NorCAS. Cases are notified from multiple sources, including antenatal ultrasound, fetal medicine departments, cytogenetic laboratories, the regional cardiology centre, pathology departments and paediatric surgery, and are included when first diagnosed at any age up to 12 years (16 years for cases born during 1985–2001). The NorCAS, as a member of the European Surveillance of Congenital Anomalies (EUROCAT [19]), follows the EUROCAT definitions, classification and inclusion criteria. Data were cross-validated with the National Congenital Anomaly System (NCAS) on an annual basis. Data on all regional births (stillbirths and live births) were provided by the UK Office for National Statistics, and TOPFAs from the NorCAS were added to the denominator.

### Inclusion and exclusion criteria

**Fig. 1:**
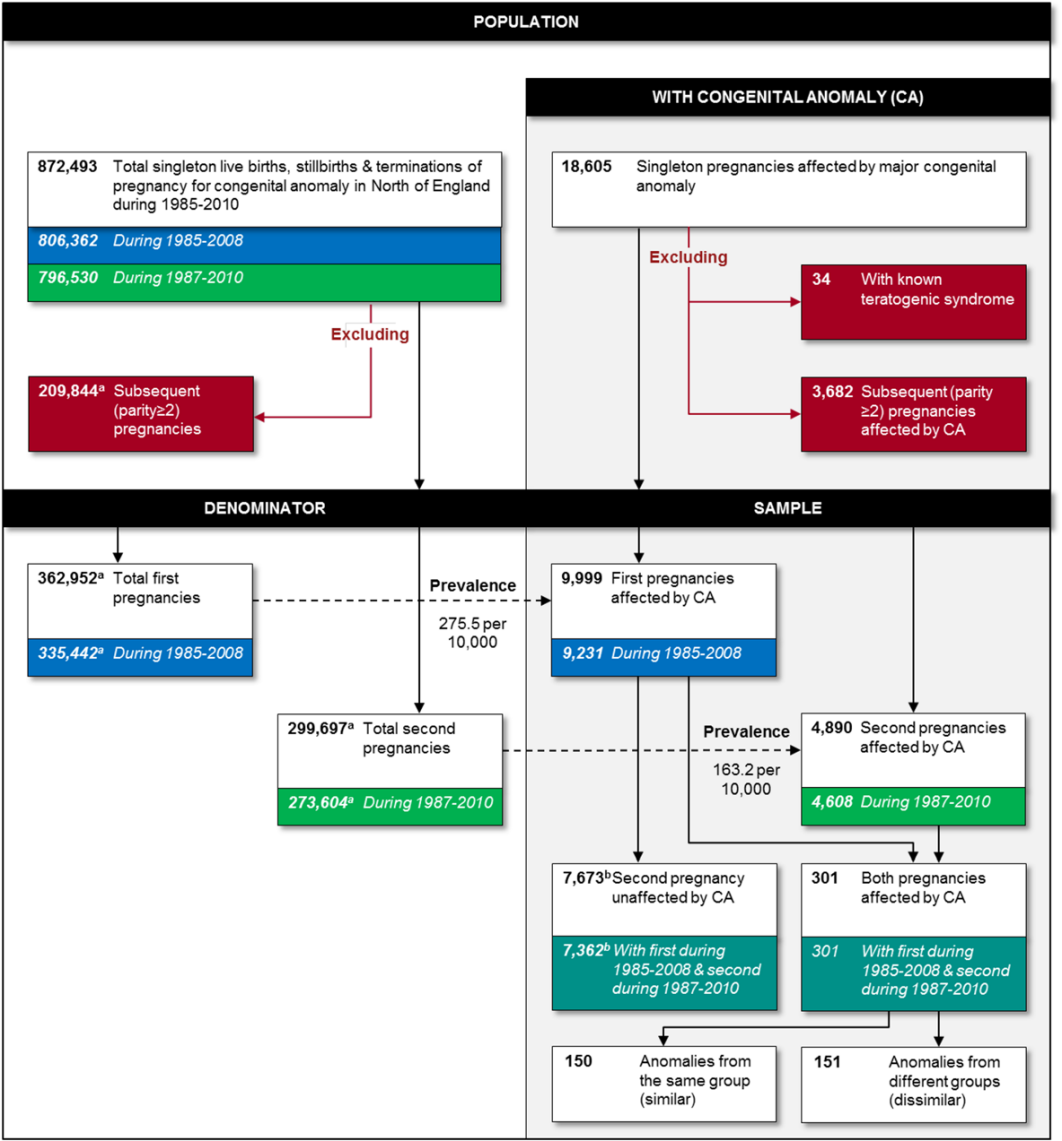
Details and derivation of the study population and sample. ^a^Estimated from the mean percentage of first and second births in England and Wales during 1990 and 2000 [27]. ^b^Estimated from the mean parity progression ratios for England and Wales during 1990 and 2000 [27]

Figure 1 shows the derivation of the study sample, which comprises all singleton first and second pregnancies affected by major congenital anomaly notified to the NorCAS during the study period. Cases arising in spontaneous miscarriage before 20 weeks, in subsequent (parity ≥2) pregnancies, that did not satisfy the EUROCAT definition of a major congenital anomaly [20] or that formed part of a known teratogenic syndrome (e.g. due to valproate use or primary cytomegalovirus infection) were excluded. Multiple pregnancies were also excluded due to higher congenital anomaly prevalence [21, 22], particularly for monochorionic twins [22].

### Identification of recurrent cases

Mothers with recurrent pregnancies affected by congenital anomaly were identified from the NorCAS maternal index number, a unique number given to each new mother when first recorded on the database. The mother’s National Health Service (NHS) number (complete from 2003 onwards), name, date of birth, postcode of residence and hospital of delivery, as well as the baby’s details were used to cross-validate all recurrent pregnancies and identify duplicate records.

### Definitions and classification of congenital anomalies

The NorCAS records text descriptions and WHO ICD-10 [23] codes for up to six individual congenital anomalies per case. These were categorised into group (the organ system affected, e.g. ‘cardiovascular’), subtype (the specific condition, e.g. ‘coarctation of the aorta’) and syndrome (e.g. ‘DiGeorge syndrome’) based on EUROCAT guidelines [24, 25]. Cases with more than one ICD code were assigned a primary diagnosis using a hierarchical approach [26] with the highest allocated from: (1) chromosomal syndromes (anomalies of chromosomal number or structure, e.g. ‘Down syndrome’); (2) genetic syndromes (patterns of anomalies arising from a single gene, e.g. ‘DiGeorge syndrome’) [24]; (3) skeletal dysplasias (syndromes of skeletal development, e.g. ‘osteogenesis imperfecta’ [24]); (4) other genetic anomalies (resulting from microdeletions or mutations, e.g. ‘neurofibromatosis’); or (5) other syndromes of non-genetic origin (recognised patterns of anomalies, with or without a known cause, e.g. ‘Noonan syndrome’) [24].

Isolated cases were allocated to their primary anomaly group and subtype. Cases with two or more structural anomalies were reviewed to identify a primary group or subtype or to assign a diagnosis of multiple anomalies (two or more unrelated structural anomalies across separate organs). More details of the classification principles are described elsewhere [26].

Congenital anomalies occurring in successive pregnancies of the same woman were considered ‘similar’ if they belonged to the same group (e.g. cardiovascular) or the same syndromic group (e.g. chromosomal syndromes), regardless of the specific subtypes, and ‘dissimilar’ if they belonged to different groups.

### Statistical analysis

The total number of first and second singleton births in the background population of the North of England during 1985–2010 (*n* = 872,493) was estimated from the mean of the percentage of first and second births in England and Wales during 1990 and 2000 [27]. Parity information for pregnancies affected by congenital anomaly, complete for around half the sample, was supplemented by multiple imputation. Ten datasets were generated via multivariate imputation by chained equations using maternal age, year of delivery, socioeconomic position (SEP, estimated from the 2007 index of multiple deprivation derived from the mother’s residential postcode at delivery) and birth weight. All statistics and standard errors, from which 95% confidence intervals (CIs) were derived, were determined independently within each imputed dataset and combined using Rubin’s rule to generate summary standard errors. The number of second pregnancies for women whose first pregnancy was affected by a congenital anomaly was predicted from the mean parity progression ratios for England and Wales during 1990 and 2000 [27].

The prevalence of congenital anomaly (overall, by group, by subtype) for first and second pregnancies was calculated as the estimated number of affected pregnancies per 10,000 (live births, stillbirths and TOPFAs). 95% CIs for prevalence proportions were approximated from the summary standard errors by logit transformation [28]. The prevalence — henceforth described as the absolute risk (AR) — of recurrent congenital anomaly (any, similar, dissimilar) in the second pregnancy was determined as the ratio of recurrent cases divided by the total estimated second pregnancies in those with a first pregnancy affected by congenital anomaly (any, by group, by subtype). Relative risks (RRs) of recurrent congenital anomaly (any, similar, dissimilar) were estimated by comparing the prevalence in those whose first pregnancy was affected by congenital anomaly (overall, by group, by subtype) to the prevalence in those with no record of congenital anomaly in their first pregnancy. To minimise any potential bias arising from the incomplete capture of linked first and second pregnancies at the beginning and end of the study period, we restricted the window for exposure, i.e. during which the first pregnancy must occur, to 1985–2008 and the window for outcome, i.e. during which the second pregnancy must occur, to 1987–2010. This corresponds with the modal inter-pregnancy interval among recurrent pregnancies (2 years) in our sample and provides a minimum of one complete year between deliveries. Only those groups and subtypes with at least three recurrent pregnancies are reported in the tables.

Summary ARs for similar and dissimilar anomalies were estimated as the weighted average of the ARs of similar (or dissimilar) anomalies across all groups, with weights equal to the number ‘exposed’ to a similar (or dissimilar) anomaly in the first pregnancy. The summary RRs for similar and dissimilar anomalies were estimated by non-linear combination of the ARs, with CIs approximated using the delta method. The effect of maternal age and SEP (analysed as tertiles) in the first pregnancy on the odds of congenital anomaly in the second pregnancy was examined by logistic regression, with non-recurrent cases weighted by the aforementioned parity progression ratios.

Two sensitivity analyses were performed. Firstly, a ‘complete case’ analysis of the AR and RR of recurrent congenital anomaly in the second pregnancy was carried out in the subsample of pregnancies with complete parity. Simple inverse probability weights were used to correct for differences in the proportion of missing data between recurrent and non-recurrent second pregnancies. Secondly, we examined the potential impact of temporal changes in the prevalence of congenital anomalies on the RR of recurrence by calculating and comparing the RRs during the first and the second half of the study period (1987–1998 and 1999–2010 respectively).

Analyses were performed using Stata version 13.1 (Statacorp, College Station, TX, USA). *P* values are presented for transparency, but no formal hypothesis tests were performed.

## Results

A total of 872,493 singleton stillbirths, live births and TOPFAs occurred during the 26 years, including 18,605 affected by major congenital anomaly, a prevalence of 213 (95% CI 210–216) per 10,000 births and TOPFAs. Of these, an estimated 9999 cases occurred in a first pregnancy from a predicted 362,952 first births and TOPFAs, a prevalence of 275 (95% CI 270–281) per 10,000, and an estimated 4890 cases occurred in a second pregnancy from a predicted 299,697 second births and TOPFAs, a prevalence of 163 (95% CI 159–168) per 10,000 (Fig. 1).

Table 1 shows the prevalence of congenital anomaly, by group and subtype, in the first and the second pregnancy individually. The estimated total prevalence of congenital anomaly was 40.8% lower (95% CI 38.7–42.7, *P* < 0.0001) among second pregnancies than first pregnancies, but there were noticeable differences in the magnitude of decrease between anomaly groups (*P* < 0.0001).

**Table 1:** Prevalence of congenital anomaly in the first and second pregnancies and relative reduction in prevalence, by group and subtype

Congenital anomaly group/subtype	First pregnancies			Second pregnancies			Relative reduction in prevalence		
	*N*	Prevalence per 10,000 (95% CI)		*N*	Prevalence per 10,000 (95% CI)		%	(95% CI)	*P* value
**Isolated anomalies**	**7162**	**197.3**	**(192.8-201.9)**	**3360**	**112.1**	**(108.4-116.0)**	**43.2**	**(40.8-45.4)**	**<0.0001**
**Nervous system**	**989**	**27.2**	**(25.6-29.0)**	**467**	**15.6**	**(14.2-17.0)**	**42.8**	**(36.2-48.8)**	**<0.0001**
Neural tube defects	649	17.9	(16.6-19.3)	309	10.3	(9.2-11.5)	42.3	(34.0-49.6)	<0.0001
Anencephaly	278	7.7	(6.8-8.6)	130	4.3	(3.7-5.2)	43.4	(30.3-54.0)	<0.0001
Encephalocele	47	1.3	(1.0-1.7)	27	0.9	(0.6-1.3)	30.4	(-11.7-56.7)	0.14
Spina bifida	324	8.9	(8.0-10.0)	152	5.1	(4.3-6.0)	43.2	(31.1-53.1)	<0.0001
Hydrocephalus	127	3.5	(2.9-4.1)	53	1.8	(1.4-2.3)	49.5	(30.4-63.3)	0.0001
Microcephaly	66	1.8	(1.4-2.3)	34	1.1	(0.8-1.6)	37.6	(5.6-58.7)	0.03
**Eye**	**81**	**2.2**	**(1.8-2.8)**	**35**	**1.2**	**(0.8-1.6)**	**47.7**	**(22.2-64.8)**	**0.002**
**Ear-face-neck**	**13**	**0.4**	**(0.2-0.6)**	**5**	**0.2**	**(0.1-0.4)**	**53.4**	**(-30.7-83.4)**	**0.15**
**Cardiovascular**	**3085**	**85.0**	**(82.1-88)**	**1619**	**54.0**	**(51.5-56.7)**	**36.4**	**(32.5-40.1)**	**<0.0001**
Transposition of the great vessels	158	4.4	(3.7-5.1)	76	2.5	(2.0-3.2)	41.7	(23.4-55.7)	0.0002
Single ventricle	22	0.6	(0.4-0.9)	11	0.4	(0.2-0.7)	39.4	(-24.9-70.6)	0.18
Ventricular septal defect (VSD)	1300	35.8	(33.9-37.8)	672	22.4	(20.8-24.2)	37.4	(31.3-43.0)	<0.0001
Atrial septal defect (ASD)	221	6.1	(5.3-6.9)	121	4.0	(3.4-4.8)	33.7	(17.2-46.9)	0.0005
Pulmonary valve stenosis	266	7.3	(6.5-8.3)	134	4.5	(3.8-5.3)	39.0	(24.9-50.4)	<0.0001
Aortic valve atresia/stenosis	133	3.7	(3.1-4.4)	73	2.4	(1.9-3.1)	33.5	(11.6-50.0)	0.006
Hypoplastic left heart	69	1.9	(1.5-2.4)	48	1.6	(1.2-2.1)	15.8	(-21.8-41.7)	0.37
Coarctation of the aorta	170	4.7	(4.0-5.4)	87	2.9	(2.3-3.6)	38.0	(19.8-52.1)	0.0004
**Respiratory**	**70**	**1.9**	**(1.5-2.4)**	**36**	**1.2**	**(0.9-1.7)**	**37.7**	**(6.9-58.3)**	**0.02**
**Orofacial clefts**	**533**	**14.7**	**(13.5-16.0)**	**189**	**6.3**	**(5.5-7.3)**	**57.1**	**(49.3-63.6)**	**<0.0001**
Cleft lip	141	3.9	(3.3-4.6)	51	1.7	(1.3-2.2)	56.2	(39.7-68.2)	<0.0001
Cleft lip and palate	206	5.7	(4.9-6.5)	71	2.4	(1.9-3.0)	58.3	(45.3-68.1)	<0.0001
Cleft palate	186	5.1	(4.4-5.9)	67	2.2	(1.8-2.9)	56.4	(42.3-67.0)	<0.0001
**Digestive system**	**496**	**13.7**	**(12.5-14.9)**	**224**	**7.5**	**(6.6-8.5)**	**45.3**	**(36.0-53.3)**	**<0.0001**
Oesophageal atresia	74	2.0	(1.6-2.6)	27	0.9	(0.6-1.3)	55.8	(31.3-71.6)	0.0004
Hirschsprung disease	60	1.7	(1.3-2.1)	24	0.8	(0.5-1.2)	51.6	(22.2-69.8)	0.003
Diaphragmatic hernia	111	3.1	(2.5-3.7)	57	1.9	(1.5-2.5)	37.8	(14.4-54.8)	0.004
**Abdominal wall**	**310**	**8.5**	**(7.6-9.5)**	**90**	**3.0**	**(2.4-3.7)**	**64.8**	**(55.5-72.2)**	**<0.0001**
Gastroschisis	243	6.7	(5.9-7.6)	65	2.2	(1.7-2.7)	67.6	(57.4-75.4)	<0.0001
**Urinary**	**1035**	**28.5**	**(26.8-30.3)**	**506**	**16.9**	**(15.5-18.4)**	**40.8**	**(34.2-46.8)**	**<0.0001**
Cystic kidney disease	199	5.5	(4.8-6.3)	103	3.4	(2.8-4.2)	37.3	(20.5-50.6)	0.0002
**Genital**	**91**	**2.5**	**(2.0-3.1)**	**39**	**1.3**	**(0.9-1.8)**	**48.1**	**(24.5-64.3)**	**0.0009**
**Limb**	**317**	**8.7**	**(7.8-9.8)**	**85**	**2.8**	**(2.3-3.5)**	**67.5**	**(58.7-74.4)**	**<0.0001**
Polydactyly	38	1.1	(0.8-1.5)	16	0.5	(0.3-0.9)	49.0	(8.6-71.6)	0.03
**Musculoskeletal**	**60**	**1.7**	**(1.3-2.1)**	**23**	**0.8**	**(0.5-1.2)**	**53.6**	**(24.9-71.3)**	**0.002**
Craniosynostosis	34	0.9	(0.7-1.3)	11	0.4	(0.2-0.7)	60.8	(22.7-80.1)	0.008
**Others**	**81**	**2.2**	**(1.8-2.8)**	**38**	**1.3**	**(0.9-1.7)**	**43.2**	**(16.5-61.4)**	**0.005**
Cystic hygroma	60	1.7	(1.3-2.1)	25	0.8	(0.6-1.2)	49.5	(19.5-68.4)	0.005
**Syndromic anomalies**	**2837**	**78.2**	**(75.4-81.1)**	**1530**	**51.1**	**(48.6-53.7)**	**34.7**	**(30.5-38.6)**	**<0.0001**
**Chromosomal syndromes**	**1488**	**41.0**	**(39.0-43.1)**	**914**	**30.5**	**(28.6-32.5)**	**25.6**	**(19.2-31.5)**	**<0.0001**
Down syndrome	741	20.4	(19.0-21.9)	450	15.0	(13.7-16.5)	26.5	(17.3-34.6)	<0.0001
Edward syndrome	134	3.7	(3.1-4.4)	109	3.7	(3.0-4.4)	1.5	(-26.8-23.5)	0.91
Klinefelter syndrome	37	1.0	(0.7-1.4)	20	0.7	(0.4-1.1)	34.5	(-12.8-62.0)	0.13
Turner syndrome	138	3.8	(3.2-4.5)	64	2.1	(1.7-2.7)	43.8	(24.5-58.2)	0.0002
**Genetic syndromes and microdeletions**	**433**	**11.9**	**(10.9-13.1)**	**213**	**7.1**	**(6.2-8.1)**	**40.4**	**(29.8-49.4)**	**<0.0001**
DiGeorge syndrome	83	2.3	(1.9-2.8)	33	1.1	(0.8-1.5)	51.8	(27.9-67.8)	0.0006
Stickler syndrome	9	0.2	(0.1-0.5)	6	0.2	(0.1-0.4)	19.3	(-126.8-71.3)	0.69
**Skeletal dysplasias**	**106**	**2.9**	**(2.4-3.5)**	**57**	**1.9**	**(1.5-2.5)**	**34.9**	**(10.1-52.8)**	**0.010**
Osteogenesis imperfecta (type II)	38	1.0	(0.8-1.4)	24	0.8	(0.5-1.2)	23.5	(-27.5-54.1)	0.31
**Other syndromes, sequences, etc.**	**296**	**8.2**	**(7.3-9.2)**	**131**	**4.4**	**(3.7-5.2)**	**46.4**	**(34.2-56.4)**	**<0.0001**
Laterality disturbance syndromes^a^	40	1.1	(0.8-1.5)	17	0.6	(0.3-0.9)	48.5	(9.2-70.8)	0.02
Noonan syndrome	17	0.5	(0.3-0.8)	9	0.3	(0.1-0.6)	35.9	(-43.8-71.4)	0.28
**Other genetic anomalies**	**54**	**1.5**	**(1.1-1.9)**	**25**	**0.8**	**(0.6-1.2)**	**43.9**	**(9.9-65.1)**	**0.02**
Ichthyosis	22	0.6	(0.4-0.9)	7	0.2	(0.1-0.5)	61.5	(9.8-83.5)	0.03
Neurofibromatosis	11	0.3	(0.2-0.6)	6	0.2	(0.1-0.4)	33.9	(-78.6-75.6)	0.42
**Multiple congenital anomalies**	**461**	**12.7**	**(11.6-13.9)**	**197**	**6.6**	**(5.7-7.5)**	**48.2**	**(38.9-56.2)**	**<0.0001**
**Any congenital anomaly**	**9999**	**275.5**	**(270.2-280.9)**	**4890**	**163.2**	**(158.7-167.8)**	**40.8**	**(38.7-42.7)**	**<0.0001**

Congenital anomaly groups (e.g. nervous system) and combined groups (i.e. isolated anomalies, syndromic anomalies and any congenital anomaly) are presented in bold text, while congenital anomaly subtypes within the groups are presented in regular text

Counts, prevalence proportions and relative reductions are the mean across ten multiply imputed datasets

Confidence intervals were estimated using a logit transformation from the summary standard errors, which were combined using Rubin’s rule

Congenital anomaly groups and subtypes are classified in accordance with EUROCAT guidelines [24, 25]

^a^Includes Ivemark syndrome, left atrial isomerism and situs inversus

From the 9231 women whose first pregnancies were affected by congenital anomaly during 1985–2008, 301 had second pregnancies affected by congenital anomaly, and a further 7362 were predicted to have a second pregnancy unaffected by congenital anomaly during 1987–2010 (Fig. 1). The AR of recurrent congenital anomaly in the second pregnancy was 408 (95% CI 365–456) per 10,000, an RR of 2.52 (95% CI 2.25–2.83, *P* < 0.0001) times greater than that among women whose first pregnancies were unaffected by congenital anomaly (Table 2). This comprised a summary AR of 204 (95% CI 169–239) per 10,000 for a similar anomaly, an RR of 23.8 (95% CI 19.6–27.9, *P* < 0.0001) times greater than that among women whose first pregnancies were unaffected, and a summary AR of 204 (95% CI 169–239) for a dissimilar anomaly, an RR of 1.40 (95% CI 1.16–1.64, *P* = 0.001) times greater than that among those whose first pregnancies were unaffected (Table 3). The RRs of a similar anomaly were substantially elevated for both syndromic (RR = 33.6, 95% CI 24.4–48.9, *P* < 0.0001) and isolated (RR = 19.9, 95% CI 15.5–24.4, *P* < 0.0001) anomalies (Table 3). For dissimilar anomalies, the RR was higher for syndromic anomalies (RR = 1.74, 95% CI 1.24–2.23, *P* = 0.004) than isolated anomalies, where the effect was very modest (RR = 1.27, 95% CI 1.00–1.55, *P* = 0.05).

**Table 2:** Absolute and relative risk of recurrent congenital anomaly (of any group) in the second pregnancy, by congenital anomaly group/subtype in the first pregnancy

Congenital anomaly group/subtype in the first pregnancy	Estimated second pregnancies^b^	Cases	Absolute risk per 10,000 (95% CI)		Relative risk (95% CI)		*P* value
**Isolated anomalies**	**5289**	**190**	**357**	**(310-645)**	**2.17**	**(1.88-2.51)**	**<0.0001**
**Nervous system**	**744**	**31**	**403**	**(283-571)**	**2.40**	**(1.69-3.42)**	**<0.0001**
Neural tube defects	482	16	332	(204-535)	1.97	(1.22-3.20)	0.007
Anencephaly	207	8	387	(193-761)	2.30	(1.15-4.57)	0.02
Encephalocele	34	2					
Spina bifida	241	6	249	(112-543)	1.48	(0.67-3.26)	0.34
Hydrocephalus	98	5	510	(213-1170)	3.03	(1.29-7.13)	0.01
Microcephaly	52	5	968	(402-2151)	5.74	(2.47-13.36)	0.0001
**Eye**	**63**	**3**	**481**	**(155-1395)**	**2.85**	**(0.94-8.65)**	**0.07**
**Ear-face-neck**	**11**	**1**					
**Cardiovascular**	**2282**	**79**	**346**	**(278-431)**	**2.07**	**(1.66-2.58)**	**<0.0001**
Transposition of the great vessels	116	2					
Single ventricle	17	2					
Ventricular septal defect (VSD)	966	38	394	(287-538)	2.35	(1.71-3.22)	<0.0001
Atrial septal defect (ASD)	165	8	487	(244-948)	2.89	(1.46-5.72)	0.003
Pulmonary valve stenosis	198	4	202	(76-531)	1.20	(0.45-3.19)	0.72
Aortic valve atresia/stenosis	100	3	301	(96-901)	1.79	(0.58-5.48)	0.31
Hypoplastic left heart	48	2					
Coarctation of the aorta	122	4	329	(123-854)	1.95	(0.74-5.17)	0.18
**Respiratory**	**50**	**1**					
**Orofacial clefts**	**398**	**14**	**352**	**(209-586)**	**2.09**	**(1.25-3.50)**	**0.006**
Cleft lip	103	4	388	(146-991)	2.30	(0.88-6.04)	0.01
Cleft lip and palate	154	6	390	(176-843)	2.32	(1.06-5.09)	0.04
Cleft palate	142	5	352	(147-820)	2.09	(0.88-4.96)	0.01
**Digestive system**	**371**	**12**	**324**	**(185-562)**	**1.93**	**(1.10-3.37)**	**0.02**
Oesophageal atresia	53	2					
Hirschsprung disease	45	2					
Diaphragmatic hernia	79	5	634	(266-1440)	3.77	(1.61-8.83)	0.003
**Abdominal wall**	**218**	**3**	**138**	**(45-419)**	**0.82**	**(0.27-2.52)**	**0.73**
Gastroschisis	172	3	174	(56-527)	1.03	(0.34-3.18)	0.96
**Urinary**	**751**	**34**	**453**	**(325-628)**	**2.70**	**(1.94-3.76)**	**<0.0001**
Cystic kidney disease	145	8	550	(277-1063)	3.27	(1.66-6.42)	0.001
**Genital**	**50**	**1**					
**Limb**	**244**	**5**	**205**	**(86-483)**	**1.22**	**(0.51-2.90)**	**0.65**
**Polydactyly**	**27**	2					
**Musculoskeletal**	**47**	**4**	**858**	**(322-2094)**	**5.09**	**(1.97-13.15)**	**0.001**
Craniosynostosis	27	2					
**Others**	**61**	**2**					
Cystic hygroma	45	2					
**Syndromic anomalies**	**2072**	**111**	**538**	**(448-645)**	**3.25**	**(2.70-3.91)**	**<0.0001**
**Chromosomal syndromes**	**1073**	**47**	**441**	**(332-583)**	**2.63**	**(1.98-3.50)**	**<0.0001**
Down syndrome	536	15	280	(169-460)	1.66	(1.01-2.75)	0.05
Edward syndrome	95	4	433	(163-1099)	2.57	(0.98-6.72)	0.06
Klinefelter syndrome	26	2					
Turner syndrome	97	4	413	(155-1055)	2.45	(0.93-6.44)	0.07
**Genetic syndromes and microdeletions**	**332**	**31**	**933**	**(663-1299)**	**5.57**	**(3.97-7.81)**	**<0.0001**
DiGeorge syndrome	61	5	822	(343-1840)	4.88	(2.09-11.40)	0.0004
Stickler syndrome	7	3	4326	(1319-7928)	25.39	(10.14-63.58)	<0.0001
**Skeletal dysplasias**	**77**	**2**					
Osteogenesis imperfecta (type II)	28	2					
**Other syndromes, sequences, etc.**	**209**	**9**	**439**	**(230-823)**	**2.61**	**(1.37-4.95)**	**0.004**
Laterality disturbance syndromes^a^	28	2					
Noonan syndrome	14	2					
**Other genetic anomalies**	**36**	**6**	**1667**	**(761-3269)**	**9.89**	**(4.72-20.73)**	**<0.0001**
Ichthyosis	15	2					
Neurofibromatosis	7	2					
**Multiple congenital anomalies**	**344**	**16**	**466**	**(287-748)**	**2.77**	**(1.71-4.48)**	**0.0001**
**Any congenital anomaly**	**7362**	**301**	**408**	**(365-456)**	**2.52**	**(2.25-2.83)**	**<0.0001**

Congenital anomaly groups (e.g. nervous system) and combined groups (i.e. isolated anomalies, syndromic anomalies and any congenital anomaly) are presented in bold text, while congenital anomaly subtypes within the groups are presented in regular text

Counts, prevalence proportions and relative risks are the mean across ten multiply imputed datasets

Confidence intervals were derived from summary standard errors, which were combined using Rubin’s rule

Congenital anomaly groups and subtypes are classified in accordance with EUROCAT guidelines [24, 25]

The risks for groups and subtypes with at least three cases of recurrent pregnancies are reported

^a^Includes Ivemark syndrome, left atrial isomerism and situs inversus

^b^Estimated second pregnancies during 1987–2010 in women with a first pregnancy during 1985–2008

**Table 3:** Absolute and relative risk of recurrent congenital anomaly in the second pregnancy for similar anomalies (i.e. from the same group) and dissimilar anomalies (i.e. from a different group), by congenital anomaly group/subtype in the first pregnancy

Congenital anomaly group/subtype in the first pregnancy	Estimated second pregnancies	From the same group (similar)						From a different group (dissimilar)					
		Cases	Absolute risk per 10,000 (95% CI)		Relative risk (95% CI)		*P* value	Cases	Absolute risk per 10,000 (95% CI)		Relative risk (95% CI)		*P* value
**Isolated anomalies**	**5289**	**91**	**172** ^**a**^	**(134-210)**	**19.90** ^**a**^	**(15.46-24.39)**	**<0.0001**	**99**	**185** ^**a**^	**(146-225)**	**1.27** ^**a**^	**(1.00-1.55)**	**0.05**
**Nervous system**	**744**	**14**	**188**	**(112-315)**	**12.41**	**(7.30-21.10)**	**<0.0001**	**17**	**215**	**(132-348)**	**1.41**	**(0.87-2.29)**	**0.17**
Neural tube defects	482	9	187	(97-355)	12.17	(6.31-23.47)	<0.0001	7	145	(69-301)	0.95	(0.45-1.98)	0.90
Anencephaly	207	3	145	(47-443)	9.33	(3.01-28.88)	0.0002	5	242	(100-572)	1.58	(0.66-3.79)	0.30
Spina bifida	241	4	166	(62-434)	10.70	(4.01-28.52)	<0.0001	2					
Hydrocephalus	98	1						4	408	(154-1040)	2.67	(1.02-6.99)	0.05
Microcephaly	52	3	581	(186-1670)	37.33	(12.25-113.7)	<0.0001	2					
**Cardiovascular**	**2282**	**45**	**197**	**(147-264)**	**3.61**	**(2.68-4.85)**	**<0.0001**	**34**	**149**	**(107-208)**	**1.33**	**(0.95-1.86)**	**0.11**
Ventricular septal defect (VSD)	966	23	238	(158-357)	4.32	(2.86-6.51)	<0.0001	15	155	(94-257)	1.38	(0.83-2.29)	0.22
Atrial septal defect (ASD)	165	6	365	(164-792)	6.56	(2.97-14.49)	<0.0001	2					
Pulmonary valve stenosis	198	3	152	(49-463)	2.72	(0.88-8.40)	0.09	1					
Coarctation of the aorta	122	3	247	(79-745)	4.42	(1.43-13.65)	0.01	1					
**Orofacial clefts**	**398**	**8**	**201**	**(101-397)**	**32.34**	**(15.98-65.43)**	**<0.0001**	**6**	**151**	**(68-332)**	**0.93**	**(0.42-2.06)**	**0.86**
Cleft lip	103	1						3	291	(94-866)	1.80	(0.59-5.49)	0.30
Cleft lip and palate	154	5	325	(136-759)	51.44	(21.39-123.7)	<0.0001	1					
Cleft palate	142	2						3	211	(68-636)	1.31	(0.43-4.01)	0.64
**Digestive system**	**371**	**4**	**108**	**(41-284)**	**14.85**	**(5.53-39.85)**	**<0.0001**	**8**	**216**	**(108-426)**	**1.34**	**(0.67-2.67)**	**0.41**
Oesophageal atresia	53	0						2					
Diaphragmatic hernia	79	2						3	381	(123-1118)	2.36	(0.78-7.19)	0.13
**Urinary**	**751**	**16**	**213**	**(131-345)**	**12.29**	**(7.50-20.15)**	**<0.0001**	**18**	**240**	**(151-378)**	**1.60**	**(1.01-2.52)**	**0.05**
Cystic kidney disease	145	5	344	(144-800)	19.41	(8.16-46.20)	<0.0001	3	206	(67-621)	1.37	(0.45-4.20)	0.58
**Limb**	**244**	**1**						**4**	**164**	**(62-429)**	**0.99**	**(0.37-2.63)**	**0.98**
**Musculoskeletal**	**47**	**0**						**4**	**858**	**(322-2094)**	**5.11**	**(1.98-13.22)**	**0.001**
**Syndromic anomalies**	**2072**	**59**	**285** ^**a**^	**(209-362)**	**33.63** ^**a**^	**(24.40-48.86)**	**<0.0001**	**52**	**253** ^**a**^	**(181-325)**	**1.74** ^**a**^	**(1.24-2.23)**	**0.004**
**Chromosomal syndromes**	**1073**	**29**	**271**	**(189-388)**	**8.74**	**(6.06-12.60)**	**<0.0001**	**18**	**170**	**(107-268)**	**1.24**	**(0.78-1.97)**	**0.35**
Down syndrome	536	7	131	(62-272)	4.11	(1.96-8.62)	0.0003	8	149	(75-296)	1.09	(0.55-2.18)	0.80
**Genetic syndromes and microdeletions**	**332**	**18**	**542**	**(344-845)**	**80.95**	**(49.98-131.1)**	**<0.0001**	**13**	**391**	**(228-663)**	**2.43**	**(1.43-4.15)**	**0.002**
DiGeorge syndrome	61	1						4	657	(247-1635)	4.08	(1.57-10.6)	0.005
Stickler syndrome	7	3	4326	(1319-7928)	590.8	(236.7-1475.0)	<0.0001	0					
**Other syndromes, sequences, etc.**	**209**	**3**	**143**	**(46-435)**	**32.61**	**(10.44-101.9)**	**<0.0001**	**6**	**296**	**(134-643)**	**1.80**	**(0.82-3.98)**	**0.15**
**Other genetic anomalies**	**36**	**5**	**1389**	**(585-2952)**	**1929.5**	**(735.7-5060.4)**	**<0.0001**	**1**					
**Multiple congenital anomalies**	**344**	**2**						**14**	**407**	**(242-677)**	**2.52**	**(1.50-4.22)**	**0.001**
**Any congenital anomaly**	**7362**	**150**	**204** ^**a**^	**(169-239)**	**23.75** ^**a**^	**(19.64-27.85)**	**<0.0001**	**151**	**204** ^**a**^	**(169-239)**	**1.40** ^**a**^	**(1.16-1.64)**	**0.001**

Congenital anomaly groups (e.g. nervous system) and combined groups (i.e. isolated anomalies, syndromic anomalies and any congenital anomaly) are presented in bold text, while congenital anomaly subtypes within the groups are presented in regular text

Counts, prevalence proportions and relative reductions are the mean across ten multiply imputed datasets

Confidence intervals were estimated using a logit transformation from the summary standard errors, which were combined using Rubin’s rule

Congenital anomaly groups and subtypes are classified in accordance with EUROCAT guidelines [24, 25]

Only those groups and subtypes with at least three cases of recurrent pregnancies are reported

^a^Estimated from the weighted average of the group-specific prevalence ratios

Table 3 also shows the extent of heterogeneity between anomaly groups in the RRs of recurrence for both similar (*P* < 0.0001) and dissimilar (*P* = 0.025) anomalies. For similar anomalies, the RRs were greatly elevated for all anomaly groups compared to women with unaffected first pregnancies. For dissimilar anomalies, most potential associations were too small — given the study sample size — to distinguish from unity, except for musculoskeletal anomalies, genetic syndromes and multiple congenital anomalies (Table 3). Despite high RRs for similar anomalies, the ARs were relatively low (Tables 3 and 4). Overall, for most major anomaly groups, absolute recurrence risks varied between 1 in 20 and 1 in 30, except for very rare genetic syndromes and other genetic anomalies for which the risks were higher (Table 4).

**Table 4:** Risk of recurrent congenital anomaly in the second pregnancy (from any group, from the same group, from a different group) presented as natural frequency, by congenital anomaly group/subtype in the first pregnancy

Congenital anomaly group/subtype in the first pregnancy	Risk, as natural frequency (95% CI)					
	Any congenital anomaly		From the same group (similar)		From a different group (dissimilar)	
**Isolated anomalies**	**1 in 28**	**(24-32)**	**1 in 58**	**(48-75)**	**1 in 54**	**(44-69)**
**Nervous system**	**1 in 25**	**(18-35)**	**1 in 53**	**(32-90)**	**1 in 47**	**(29-68** ^**b**^ **)**
Neural tube defects	1 in 30	(19-49)	1 in 54	(28-103)	1 in 65^a^	(33-65^b^)
Anencephaly	1 in 26	(13-52)	1 in 69	(23-215)	1 in 41	(17-63^b^)
Spina bifida	1 in 40	(18-61^b^)	1 in 60	(23-161)		
Hydrocephalus	1 in 20	(9-47)			1 in 25	(10-62^b^)
Microcephaly	1 in 10	(5-25)	1 in 17	(6-54)		
**Cardiovascular**	**1 in 29**	**(23-36)**	**1 in 51**	**(38-68)**	**1 in 67** ^a^	**(48-92)**
Ventricular septal defect (VSD)	1 in 25	(19-35)	1 in 42	(28-63)	1 in 64^a^	(39-71^b^)
Atrial septal defect (ASD)	1 in 21	(11-41)	1 in 27	(13-61)		
Pulmonary valve stenosis	1 in 49	(19-61^b^)	1 in 66	(22-205)		
Aortic valve atresia/stenosis	1 in 33	(11-61^b^)	1 in 50	(13-201)		
Coarctation of the aorta	1 in 30	(12-61^b^)	1 in 41	(13-127)		
**Orofacial clefts**	**1 in 28**	**(17-48)**	**1 in 50**	**(25-99)**	**1 in 64** ^**a**^	**(30-64** ^**b**^ **)**
Cleft lip	1 in 26	(10-61^b^)			1 in 34	(12-62^b^)
Cleft lip and palate	1 in 26	(12-57)	1 in 31	(13-74)		
Cleft palate	1 in 28	(12-61^b^)			1 in 47	(16-62^b^)
**Digestive system**	**1 in 31**	**(18-54)**	**1 in 93**	**(35-246)**	**1 in 46**	**(23-64** ^**b**^ **)**
Diaphragmatic hernia	1 in 16	(7-38)			1 in 26	(9-62^b^)
**Abdominal wall**	**1 in 61** ^**a**^	**(24-61** ^**b**^ **)**			**1 in 62** ^**a**^	**(28-62** ^**b**^ **)**
Gastroschisis	1 in 57	(19-61^b^)			1 in 62^a^	(22-62^b^)
**Urinary**	**1 in 22**	**(16-31)**	**1 in 47**	**(29-76)**	**1 in 42**	**(26-66** ^**b**^ **)**
Cystic kidney disease	1 in 18	(9-36)	1 in 29	(12-70)	1 in 48	(16-63^b^)
**Limb**	**1 in 49**	**(21-61** ^**b**^ **)**			**1 in 61** ^**a**^	**(23-62** ^**b**^ **)**
Polydactyly	1 in 14	(4-55)			1 in 14	(4-55)
**Musculoskeletal**	**1 in 12**	**(5-31)**			**1 in 12**	**(5-31)**
**Syndromic anomalies**	**1 in 19**	**(15-22)**	**1 in 35**	**(28-48)**	**1 in 40**	**(31-55)**
**Chromosomal syndromes**	**1 in 23**	**(17-30)**	**1 in 37**	**(26-53)**	**1 in 59**	**(37-75** ^**b**^ **)**
Down syndrome	1 in 36	(22-59)	1 in 77	(37-160)	1 in 67^a^	(34-67^b^)
Edward syndrome	1 in 23	(9-61)				
Turner syndrome	1 in 24	(9-61^b^)				
**Genetic syndromes and microdeletions**	**1 in 11**	**(8-15)**	**1 in 18**	**(12-29)**	**1 in 26**	**(15-44)**
DiGeorge syndrome	1 in 12	(5-29)			1 in 15	(6-40)
Stickler syndrome	1 in 2	(1-8)	1 in 2	(1-8)		
**Other syndromes, sequences, etc.**	**1 in 23**	**(12-43)**	**1 in 70**	**(23-216)**	**1 in 34**	**(16-63** ^**b**^ **)**
**Other genetic anomalies**	**1 in 6**	**(3-13)**	**1 in 7**	**(3-17)**		
**Multiple congenital anomalies**	**1 in 21**	**(13-35)**			**1 in 25**	**(15-41)**
**Any congenital anomaly**	**1 in 24**	**(22-27)**	**1 in 49**	**(42-59)**	**1 in 49**	**(42-59)**

Congenital anomaly groups (e.g. nervous system) and combined groups (i.e. isolated anomalies, syndromic anomalies and any congenital anomaly) are presented in bold text, while congenital anomaly subtypes within the groups are presented in regular text

^a^Estimate truncated at baseline risk

^b^Confidence limit truncated at baseline risk

Only those groups and subtypes with at least three cases of recurrent pregnancies are reported

The odds of recurrence were higher in women with more deprived SEPs (odds ratio, OR = 1.48, 95% CI 1.04–2.10, *P* = 0.03, for most vs least deprived tertile) and declined with increasing maternal age at delivery (OR = 0.95, 95% CI 0.92–0.98, *P* < 0.0001 per year increase). However, maternal age and SEP were highly correlated, and when both were examined concurrently, the effect of SEP was attenuated (SEP: adjusted OR, aOR, for most vs least deprived tertile = 1.18, 95% CI 0.82–1.72, *P* = 0.37; maternal age: aOR, per year = 0.95, 95% CI 0.93–0.98, *P* = 0.001).

When the analysis was performed in the subsample of pregnancies with complete parity, the AR of recurrent congenital anomaly was 419 per 10,000 (95% CI 354–492), and the RR was 2.45 (95% CI 2.09–2.88, both within ± 3% of the estimates derived using multiple imputation (AR = 408 per 10,000 (95% CI 365–456) and RR = 2.52 (95% CI 2.25–2.83) respectively).

There was no evidence that the RR of recurrence had changed over the study period, with similar RRs of 2.46 (95% CI 2.06–2.94) during 1987–1998 and 2.54 (95% CI 2.18–2.96) during 1999–2010 (*P* = 0.89).

## Discussion

Using population-based register data, we found that history of congenital anomaly in the first pregnancy was associated with a 2.5-fold risk of recurrent anomaly in the second pregnancy despite lower overall prevalence of a congenital anomaly in second pregnancies compared with first pregnancies. For similar anomalies, the recurrence risk was nearly 24 times higher, while for dissimilar anomalies, the increase was considerably more modest (1.4-fold). For similar anomalies, much higher recurrence risks were observed for both isolated (20-fold) and syndromic (34-fold) groups of congenital anomalies. For dissimilar anomalies, the increased risk of recurrence was much lower for both isolated (1.3-fold) and syndromic (1.7-fold) anomalies. Absolute recurrence risks varied between 1 in 20 and 1 in 30 for most major anomaly groups. Women from the most deprived areas experienced nearly 1.5 times higher odds of recurrence than those from the least deprived areas, although this was partly explained by differences in the maternal age distributions.

This is the first study to estimate the absolute and relative recurrence risks of major congenital anomalies in siblings using population-based data derived from a high-quality congenital anomaly register, which employs consistent, internationally approved and clinically meaningful definition, classification and inclusion criteria. The NorCAS receives information, regardless of pregnancy outcome, from multiple sources up to 12 years after birth, using a well-established network of local clinicians, and thereby assures high case ascertainment.

This study has some limitations. Despite being the largest study of its kind, the absolute numbers of recurrent anomalies were low. Our estimates are thus relatively uncertain for some rare groups and subtypes, while others could not be explored. Due to the rarity of the outcomes under examination, and the large number explored, we did not perform classical hypothesis tests, as the division into ‘statistically significant’ and otherwise would have been potentially misleading. To minimise any potential bias arising from the incomplete capture of linked first and second pregnancies, we restricted first pregnancies to 1985–2008 and second pregnancies to 1987–2010, providing a minimum of one complete year between the first and the second delivery. This approach should have improved the accuracy of our estimates at the expense of some precision, due to the small loss of sample size. The most recent second pregnancies will have experienced shorter postnatal follow-up periods than earlier births, potentially resulting in lower rates of congenital anomaly. However, more than 95% of major congenital anomalies are typically diagnosed either prenatally or within the first 2 years of life, and even babies born at the end of 2010 will have been followed for a minimum of 3 years, since our study includes notifications until the end of 2013. Regardless, we found no difference in the RR of recurrence between pregnancies delivered during 1987–1998 and 1999–2010, suggesting that temporal factors, such as improved diagnosis and ascertainment, are not related to the mechanisms underlying the increased risk of recurrence. The RR of recurrence may be slightly overestimated, as our denominator did not include late miscarriages, which may be more common in women with a history of congenital anomaly in a previous pregnancy. However, any such effect is likely small, since the proportions of late miscarriages in recurrent and non-recurrent groups are similarly rare, at around 1% of all observed deliveries.

Parity information was incomplete for around half of the pregnancies complicated by congenital anomaly. To minimise the risk of potential bias, we imputed parity from maternal age, SEP, birth weight and year of delivery, all of which were correlated with parity in our sample. For comparison, we also estimated the ARs and RRs of recurrence in those with complete parity information using simple probability weights to correct for differences in the proportion of missing data between recurrent and non-recurrent second pregnancies. The similarity of the estimates produced by these two approaches (within ± 3%) provides reassurance of the performance of the imputation. Parity data were also missing for the background population, so the proportion of first and second pregnancies required estimation from UK national trends, as did the number of second pregnancies for women with a history of congenital anomaly in the first pregnancy. Our estimate of the parity distribution is likely to be very close to the true value, as parity progression ratios are remarkably stable. For every year between 1985 and 2000, for example, the first-to-second pregnancy parity progression ratio for England and Wales has varied from a low of 0.78 to a high of 0.81 [27]. More recent data on the birth order distribution in England and Wales suggest that this has remained equally static (exactly 0.79 in 2009, 2010 and 2011) [29]. Although parity progression ratios do vary by maternal age and SEP, which may lead to some regional variation, the effect sizes are modest, and any divergence from the national profile is therefore likely to be modest [30]. In the absence of conflicting evidence, we assumed that women whose first pregnancy was affected by congenital anomaly had the same probability of second pregnancy as the general population, but experience of congenital anomaly is plausibly associated with altered reproductive health and/or behaviour. In a Dutch sample of parents who experienced termination of pregnancy for congenital anomaly, 6% of mothers and 9% of fathers indicated they ‘would refrain from a next pregnancy for fear of another anomalous child’ [31]. If we recalculate our RR for recurrence using a similar 9% decrease in the probability of a subsequent pregnancy, it increases to 2.78 (95% CI 2.47–3.12). Similarly, using an even smaller probability of parity progression, like the 68% observed in Norway during 1967–1989 after a first pregnancy affected by congenital anomaly [16], increases the RR further; in this instance to 2.99 (95% CI 2.66–3.36). For our RR to be an overestimate of the true RR, experience of congenital anomaly in the first pregnancy would need to be associated with a higher probability of subsequent pregnancy. The RR plateaus at 2.00 (1.78–2.25) if 100% of such women are assumed to have had a second pregnancy. It is hence unlikely that the results or implications of our study are materially biased by the use of an estimated parity progression ratio.

Our finding of a lower prevalence of congenital anomalies in the second pregnancy compared with the first pregnancy agrees with a previous study suggesting that nulliparity is associated with a higher risk of specific congenital anomalies [32]. There are few population-based studies exploring recurrence risk of unselected congenital anomalies in singleton siblings. Previous studies from Scandinavia [15, 16] and the USA [17] restricted their analysis of recurrence risk to first and second births to avoid issues related to selective fertility [33]. We followed a similar approach. The summary RR of recurrence estimated in our study (RR = 2.5) is comparable with that reported in the Norwegian study [16] (RR = 2.4) and slightly higher than that in the US [17] (RR = 1.9) and in the Danish [15] (RR = 1.8) studies. The summary RR for similar anomalies was higher in our study (23.7-fold) than in any of these studies: RR = 11.7, RR = 7.6 and OR = 5.4 in the US [17], Norwegian [16] and Danish [15] studies respectively. This likely results from better ascertainment and our use of the EUROCAT classification system, which includes much more homogeneous groupings. For dissimilar anomalies, the RR was similarly low across all studies, including ours, ranging between 1.2 and 1.5. Between-study comparisons of specific anomaly groups are particularly hindered by differences in classification and inclusion criteria. In many of the earlier studies, for example, chromosomal syndromes were combined with other syndromes, yet we found heterogeneity between these groups. These issues preclude detailed comparison with an older and much smaller UK study, which used data from the Birmingham Malformation Register for 1964–1984 [34]; the overall RRs of 17.8 and 1.8 for similar and dissimilar anomalies respectively are however remarkably similar. In summary, our study has a number of strengths over all existing population-based studies which were published in the 1990s and hence used old classification schemes of congenital anomalies, applied inconsistent inclusion criteria (including minor anomalies) and lacked detail, particularly with respect to absolute risks, for many specific congenital anomaly groups and subtypes. Our risk estimates are generalizable to other regions of the UK and Europe.

The causes of some congenital anomalies (e.g. single gene defects, chromosomal anomalies, specified syndromes) are known, and the recurrence risks can be estimated accordingly. However, for about 70% of non-syndromic structural anomalies (e.g. cardiovascular anomalies, neural tube defects and orofacial clefts), the causes are still unknown and are thought to consist of a multifactorial combination of genetic factors, environmental factors and gene-environment interaction [3, 35, 36]. Some genetic variants, both common and rare, have been implicated in the risk of non-syndromic congenital anomalies [36]. Polymorphisms in the folate-related gene *MTHFR*, for example, have been shown to increase the risk of neural tube defects [36]. For similar anomalies in the second pregnancy, we estimated 12.4-fold, 3.6-fold and 32.3-fold increased recurrence risks for nervous system anomalies (including neural tube defects), cardiovascular anomalies and orofacial clefts respectively. These are many times greater than the effect of most recognised environmental teratogens, suggesting that a specific mechanism, such as a genetic or epigenetic exposure, may be involved — especially given the consistency in the estimated RRs across different studies. Repeated environmental exposures such as maternal diabetes, maternal obesity and micronutrient deficiencies, are however also likely to contribute to the risk of recurrence. We found that women from more deprived SEPs experienced a higher risk of recurrent congenital anomaly in the second pregnancy than those from more advantaged SEPs, although the effect was attenuated by adjustment for maternal age. Younger maternal age is strongly correlated with a more deprived SEP, which likely explains the dilution of the area-based measure. Potential explanations for the higher risk of recurrence in younger women include various behaviour risk factors such as smoking, recreational drug use, alcohol consumption and unplanned pregnancy, none of which could be controlled for in our study. Similarly, the reliance on routinely collected data meant we were unable to explore other potential modifiers of the risk of recurrence, such as a change in partner and/or residence between pregnancies.

## Conclusions

This study provides detailed information on the ARs and RRs of recurrence of congenital anomaly for several specific (and well-defined) groups and subtypes for women whose first pregnancies were complicated by congenital anomaly. This should prove extremely useful during pre-conception counselling of mothers and their families when considering subsequent pregnancies and during antenatal or postnatal counselling to support decision making and — in many cases — provide reassurance [13]. Although the relative risks of recurrence were alarmingly high for many groups and subtypes – particularly for similar anomalies where unidentified genetic factors may be involved – the absolute risks, in the vast majority of instances, are relatively low. For structural anomalies, like neural tube defects, these could be further reduced by help with planning and preparation for pregnancy, particularly in higher risk groups such as women with diabetes [37]. Furthermore, many potential pre-pregnancy interventions, such as maternal and paternal [36] periconception folate supplementation, review of current medication and help and advice with diet and physical activity during preparation for next pregnancy, are likely to reduce the risk of other adverse pregnancy outcomes, including congenital anomalies.

## Abbreviations

AR: Absolute risk
CI: Confidence interval
EUROCAT: European Surveillance of Congenital Anomalies
NorCAS: Northern Congenital Abnormality Survey
RR: Relative risk
SEP: Socioeconomic position
TOPFA: Termination of pregnancy for fetal anomaly

## References

[1] British Isles Network of Congenital Anomaly Registers (BINOCAR). Congenital anomaly statistics 2012 England and Wales. http://www.binocar.org/content/Annual%20report%202012_FINAL_nologo.pdf. Accessed 1 Apr 2015.

[2] Centers for Disease Control and Prevention (CDC) (2008). Update on overall prevalence of major birth defects - Atlanta, Georgia, 1978-2005. MMWR Morb Mortal Wkly Rep.

[3] Christianson A, Howson CP, Modell B (2006). March of Dimes global report on birth defects: the hidden toll of dying and disabled children.

[4] Dolk H, Loane M, Garne E (2010). The prevalence of congenital anomalies in Europe. Adv Exp Med Biol..

[5] Murphy SL, Xu J, Kochanek KD (2013). Deaths: final data for 2010. Natl Vital Stat Rep..

[6] Office for National Statistics. Childhood, infant and perinatal mortality in England and Wales. 2010. http://www.ons.gov.uk/ons/dcp171778_263357.pdf. Accessed 7 Apr 2015.

[7] Word Health Organization. Birth defects. Report by the Secretariat for Sixty-Third World Health Assembly. 2010. http://apps.who.int/gb/ebwha/pdf_files/WHA63/A63_10-en.pdf. Accessed 8 May 2015.

[8] CDC’s National Center on Birth Defects and Developmental Disabilities. Strategic plan 2011-2015. 2011. http://www.cdc.gov/NCBDDD/AboutUs/documents/NCBDDD_StrategicPlan_2-10-11.pdf. Accessed 2 June 2015.

[9] Boyd PA, Devigan C, Khoshnood B, Loane M, Garne E, Dolk H, Group EW (2008). Survey of prenatal screening policies in Europe for structural malformations and chromosome anomalies, and their impact on detection and termination rates for neural tube defects and Down’s syndrome. BJOG..

[10] Asplin N, Wessel H, Marions L, Georgsson OS (2014). Pregnancy termination due to fetal anomaly: women’s reactions, satisfaction and experiences of care. Midwifery..

[11] Aalfs CM, Mollema ED, Oort FJ, de Haes JC, Leschot NJ, Smets EM (2004). Genetic counseling for familial conditions during pregnancy: an analysis of patient characteristics. Clin Genet..

[12] Wille MC, Weitz B, Kerper P, Frazier S (2004). Advances in preconception genetic counseling. J Perinat Neonatal Nurs..

[13] Harper PS (2011). Practical genetic counselling.

[14] WHO Media Centre. Fact sheet N°370. Congenital anomalies. http://www.who.int/mediacentre/factsheets/fs370/en/. Accessed 2 June 2015.

[15] Basso O, Olsen J, Christensen K (1999). Recurrence risk of congenital anomalies—the impact of paternal, social, and environmental factors: a population-based study in Denmark. Am J Epidemiol..

[16] Lie RT, Wilcox AJ, Skjaerven R (1994). A population-based study of the risk of recurrence of birth defects. N Engl J Med..

[17] Mueller BA, Schwartz SM (1997). Risk of recurrence of birth defects in Washington State. Paediatr Perinat Epidemiol..

[18] Office for National Statistics. Ethnicity and national identity in England and Wales. 2011. http://www.ons.gov.uk/peoplepopulationandcommunity/culturalidentity/ethnicity/articles/ethnicityandnationalidentityinenglandandwales/2012-12-11#ethnicity-across-the-english-regions-and-wales. Accessed 24 May 2016.

[19] European Surveillance of Congenital Anomalies (EUROCAT). http://www.eurocat-network.eu/. Accessed 6 Oct 2016.

[20] European Surveillance of Congenital Anomalies (2013). Chapter 3.2: Minor anomalies for exclusion. In: EUROCAT Guide 14 and reference documents.

[21] Li SJ, Ford N, Meister K, Bodurtha J (2003). Increased risk of birth defects among children from multiple births. Birth Defects Res A Clin Mol Teratol..

[22] Glinianaia SV, Rankin J, Wright C (2008). Congenital anomalies in twins: a register-based study. Hum Reprod..

[23] World Health Organization (2010). Congenital malformations, deformations and chromosomal abnormalities (Q00-Q99). International statistical classification of diseases and related health problems: 10th revision.

[24] European Surveillance of Congenital Anomalies. EUROCAT syndrome guide: definition and coding of syndromes. www.eurocat-network.eu/content/EUROCAT-Syndrome-Guide-6-2008.pdf. Accessed 12 Feb 2014.

[25] European Surveillance of Congenital Anomalies (2013). Chapter 3.3: EUROCAT subgroups of congenital anomalies (version 2014). EUROCAT Guide 14 and reference documents.

[26] Tennant PW, Pearce MS, Bythell M, Rankin J (2010). 20-year survival of children born with congenital anomalies: a population-based study. Lancet..

[27] Smallwood S. New estimates of trends in births by birth order in England and Wales. Popul Trends. 2002;Summer:32–48. 12138613

[28] Sukasih A, Jang D (2005). An application of confidence interval methods for small proportions in the Health Care Survey of DoD Beneficiaries. Proceedings of the Survey Research Methods Section, American Statistical Association 2005.

[29] Office for National Statistics (Demographic Analysis Unit). Parity distribution by age estimates, 2009-2011 and partial 2012. www.ons.gov.uk/ons/about-ons/business-transparency/freedom-of-information/what-can-i-request/published-ad-hoc-data/pop/june-2014/live-births--number-of-previous-live-born-children.xls. Accessed 23 May 2016.

[30] Rendall M, Smallwood S (2003). Higher qualifications, first birth timing, and further childbearing in England and Wales. Popul Trends..

[31] Korenromp MJ, Page-Christiaens GC, van den Bout J, Mulder EJ, Hunfeld JA, Potters CM, Erwich JJ, van Binsbergen CJ, Brons JT, Beekhuis JR (2007). A prospective study on parental coping 4 months after termination of pregnancy for fetal anomalies. Prenat Diagn..

[32] Duong HT, Hoyt AT, Carmichael SL, Gilboa SM, Canfield MA, Case A, McNeese ML, Waller DK (2012). National Birth Defects Prevention Study. Is maternal parity an independent risk factor for birth defects?. Birth Defects Res A Clin Mol Teratol.

[33] Skjaerven R, Wilcox AJ, Lie RT, Irgens LM (1988). Selective fertility and the distortion of perinatal mortality. Am J Epidemiol..

[34] Knox EG, Lancashire RJ (1991). Familial concordances in twins and sibs. Epidemiology of congenital malformations.

[35] Weinhold B (2009). Environmental factors in birth defects: what we need to know. Environ Health Perspect..

[36] Webber DM, MacLeod SL, Bamshad MJ, Shaw GM, Finnell RH, Shete SS, Witte JS, Erickson SW, Murphy LD, Hobbs C (2015). Developments in our understanding of the genetic basis of birth defects. Birth Defects Res A Clin Mol Teratol..

[37] Glinianaia SV, Tennant PW, Crowder D, Nayar R, Bell R (2014). Fifteen-year trends and predictors of preparation for pregnancy in women with pre-conception Type 1 and Type 2 diabetes: a population-based cohort study. Diabet Med..

